# Predicting High-Strength Concrete’s Compressive Strength: A Comparative Study of Artificial Neural Networks, Adaptive Neuro-Fuzzy Inference System, and Response Surface Methodology

**DOI:** 10.3390/ma17184533

**Published:** 2024-09-15

**Authors:** Tianlong Li, Jianyu Yang, Pengxiao Jiang, Ali H. AlAteah, Ali Alsubeai, Abdulgafor M. Alfares, Muhammad Sufian

**Affiliations:** 1 School of Civil Engineering, Changsha University of Science & Technology, Changsha 410000, Hunan, China; 2 Qionghai Construction Engineering Quality and Safety Supervision Station, Qionghai 571442, Hainan, China; 3 China Construction Fifth Engineering Division Corp., Ltd., Changsha 410000, China; 4 Department of Civil Engineering, College of Engineering, University of Hafr Al Batin, Hafr Al Batin 39524, Saudi Arabia; ali.alateah@uhb.edu.sa; 5 Department of Civil Engineering, Jubail Industrial College, Royal Commission of Jubail, Jubail Industrial City 31961, Saudi Arabia; subeaia@rcjy.edu.sa; 6 Department of Electrical Engineering, College of Engineering, University of Hafr Al Batin, Hafr Al Batin 39524, Saudi Arabia; aalfares@uhb.edu.sa; 7 School of Civil Engineering, Southeast University, Nanjing 210096, China

**Keywords:** high-strength concrete, compressive strength, sensitivity analysis, artificial neural networks, neuro-fuzzy inference systems, central composite design

## Abstract

Machine learning and response surface methods for predicting the compressive strength of high-strength concrete have not been adequately compared. Therefore, this research aimed to predict the compressive strength of high-strength concrete (HSC) using different methods. To achieve this purpose, neuro-fuzzy inference systems (ANFISs), artificial neural networks (ANNs), and response surface methodology (RSM) were used as ensemble methods. Using an ANN and ANFIS, high-strength concrete (HSC) output was modeled and optimized as a function of five independent variables. The RSM was designed with three input variables: cement, and fine and coarse aggregate. To facilitate data entry into Design Expert, the RSM model was divided into six groups, with *p*-values of responses 1 to 6 of 0.027, 0.010, 0.003, 0.023, 0.002, and 0.026. The following metrics were used to evaluate model compressive strength projection: R, R^2^, and MSE for ANN and ANFIS modeling; R^2^, Adj. R^2^, and Pred. R^2^ for RSM modeling. Based on the data, it can be concluded that the ANN model (R = 0.999, R^2^ = 0.998, and MSE = 0.417), RSM model (R = 0.981 and R^2^ = 0.963), and ANFIS model (R = 0.962, R^2^ = 0.926, and MSE = 0.655) have a good chance of accurately predicting the compressive strength of high-strength concrete (HSC). Furthermore, there is a strong correlation between the ANN, RSM, and ANFIS models and the experimental data. Nevertheless, the artificial neural network model demonstrates exceptional accuracy. The sensitivity analysis of the ANN model shows that cement and fine aggregate have the most significant effect on predicting compressive strength (45.29% and 35.87%, respectively), while superplasticizer has the least effect (0.227%). RSME values for cement and fine aggregate in the ANFIS model were 0.313 and 0.453 during the test process and 0.733 and 0.563 during the training process. Thus, it was found that both ANN and RSM models presented better results with higher accuracy and can be used for predicting the compressive strength of construction materials.

## 1. Introduction

Long-span bridges, high-rise structures, and other specialist projects frequently utilize high-strength concrete (HSC), which is characterized by high density, low porosity, and high compressive strength (CS) [1]. Compact density, low permeability, remarkable mechanical strength, and significant durability are all exhibited by the HSC composite. It also exceeds traditional concrete in terms of performance and homogeneity standards [2]. High-rise structures, on the other hand, can benefit from HSC since it allows for bigger column spacing and more floor area to be achieved without taking away from lower levels [3]. Various researchers have examined the performance of connectors, including their shear resistance and ductility when they are integrated into the HSC [4]. An analysis was conducted to examine the overall performance of HSC beams, focusing on factors such as mid-span deflection, failure mechanism, and crack propagation [5]. Along with several cutting-edge technologies, like 3D printing and building information modeling, HSC can also be used as the primary building material [6]. Solid waste elements like waste glass and reclaimed aggregate can also be used in HSC to offset waste deficiencies [7]. As a result, the combination of HSC and solid wastes offers the advantages of increased strength and prospective sustainability [8]. HSC has a significant carbon footprint since it contains 400–700 kg/m^3^ of Portland cement. Cement clinker production uses 10 MJ of energy and releases 1.2 tons of CO_2_ [9]. Traditional evaluation methods like nonlinear regression and linear regression have predicted the compressive strength of the HSC composite. Even with simple regression models, reliable predictions are difficult; therefore, advanced methods are in demand [10].

The independent–dependent relationship for mechanical properties can be predicted in several ways [11]. RSM and ANN are two prominent approaches to showing interrelationships [12]. RSM (response surface methodology) and ANN (artificial neural network) approaches are commonly employed for predicting and simulating the properties of concrete materials [13]. Many recent studies have shown that an ANN can solve engineering problems. However, data for forecasting concrete compressive strength is sometimes complex or insufficient [14]. The compressive strength of self-compacting concrete (SCC) including bottom ash was predicted by Siddique et al. [15] using artificial neural networks. Artificial neural network (ANN) models are gradually replacing classic linear models for forecasting concrete properties [16]. ANN models have been utilized in numerous studies to simulate systems that replace sand with fly ash [17,18], silica fume [19,20], metakaolin, and blast furnace slag [20]. Support vector machines (SVM) [17], back-propagation neural networks (BPNNs) [21], genetic programming (GEP) [20], multi-layer perceptron neural networks (MLP-ANN) [22], and artificial neural networks with genetic algorithms (GA-ANN) [19] are just a few of the many other architectures that have also been studied. ANN outperformed XGBoost in recognizing waste marble powder (WMP) content. The XGBoost and ANN models optimized concrete WMP dosages [23]. A lengthy experiment examined whether waste quartz mineral dust (WQMD) [24], supplementary cementitious material (SCM), and waste sawdust (WS) [25] could produce high-efficiency lightweight concrete without cement.

Adaptive neuro-fuzzy inference systems and artificial neural networks (ANNs) are two examples of how computer technology has made it possible to tackle engineering problems more accurately and effectively [26]. The ANFIS is built upon the Takagi–Sugeno fuzzy inference system, which combines fuzzy logic and neural networks [27]. Lab testing and intricate analysis are not necessary for forecasting when using soft computing techniques like ANN and ANFIS [28]. To forecast FRP sheet–concrete bond strength under direct tension, Ref. [29] created a hybrid model employing the ant colony optimization (ACO) algorithm and fuzzy c-means (FCM) clustering method and integrated it into the adaptive neuro-fuzzy inference system (ANFIS). The compressive strength of brick–mortar masonry was studied by Mishra et al. [30] using ANN and ANFIS. The association between concrete strength and mixing percentage was examined using ANFIS [31].

Response surface methodology (RSM) is a useful statistical analysis technique that is especially beneficial for experimental research. Using a restricted number of trials enables researchers to investigate the mathematical relationship between input and output variables [32]. The response surface methodology (RSM), a collection of statistical and mathematical tools, is valuable and effective in modeling and evaluating experimental issues [33]. The RSM was used to optimize concrete made with crumb rubber (CR) fine aggregate and metakaolin (MK) cement replacement. Compressive strength was negatively affected by CR [34]. Using response surface methodology (RSM), Ref. [35] evaluated how the self-compacting concrete (SCC) mix parameter affected slump flow, filling capacity, V-funnel flow duration, and compressive strength of fresh and hardened characteristics. RSM was used in fly ash and metakaolin high-performance concrete to increase compressive strength and reduce water sorptivity, water absorption, and chloride permeability [36]. Response surface methodology (RSM) predicts desired concrete qualities. Using mathematical and statistical models and analysis of variance (ANOVA) statistics to assess how a factor impacts observed responses, the best key component mix was identified [37]. Using palm oil clinker (POC) and nano palm oil fuel ash (POFA), the RSM improved lightweight concrete. POC decreased tensile, flexure, and ultrasonic pulse velocity (UPV) with nano palm oil ash [38]. Hacene [39] used RSM and ANN to estimate concrete compressive strength probabilistically and found that RSM or ANN modeling is practicable and promising depending on its objectives.

## 2. Research Significance

Much of the previous literature emphasized numerous methods for forecasting the compressive strength (CS) of high-strength concrete (HSC). Still, it became clear to us that there is a deficiency in comparing machine learning and non-machine learning methods such as RSM, ANN, and ANFIS. This study employs ANN, RSM, and ANFIS to predict the compressive strength and to analyze the sensitivity ranking of various affecting factors on high-strength concrete (HSC) outcomes. The Levenberg–Marquardt approach was used by ANN, whilst central composite design (CCD) was used by RSM, in the comparison between ANN, AFNIS, and RSM. This study’s final goal is sensitivity analysis for the ANN and AFNIS models. This groundbreaking work provides a new way to estimate the mechanical strength of HSC for use in sophisticated engineering applications.

## 3. Experimental Dataset

**Figure 1:**
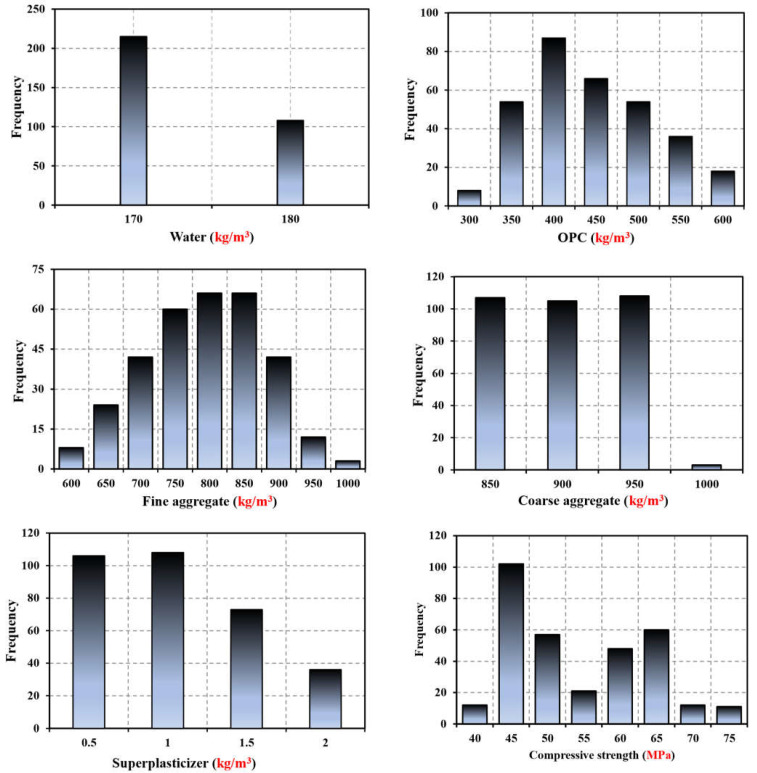
Five independent factors and concrete compressive strength histograms.

To construct the prediction model, a substantial quantity of empirical data on the compressive strength of concrete is necessary. For this reason, we have compiled a list of 324 concrete compressive strength tests from published research [40,41]. The five components of the tested concrete are fine aggregate (FA), ordinary Portland cement (OPC), superplasticizer (SP), water, and coarse aggregate (CA). There are six parameters in all the experimental datasets. The experimental parameters are listed in Table 1 together with their name, unit, mean, standard deviation (SD), and lowest and maximum values. Furthermore, Figure 1 plots the statistical distribution of the relevant parameters, which enables us to observe the parameters directly.

The input and output variables must be identified before the learning process begins. Concrete’s ultimate compressive strength is influenced by its constituents. As a result, Table 1 displays the total number of input variables (X_1_, X_2,_ …, X_5_) and output variables (Y) considered in this study.

### Dataset Sensitivity Analysis and Statistical Description

**Figure 2:**
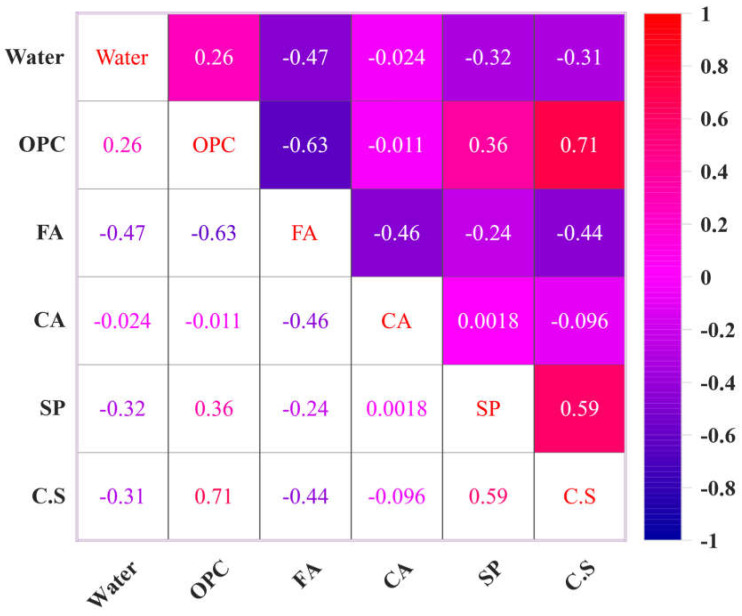
Comparison of experimental datasets using Pearson’s correlation coefficient.

The dataset, which consisted of 324 data samples, was analyzed in this section to develop and test the proposed ANN, RSM, and ANFIS models in this study. Conditions include fine aggregate (FA), ordinary Portland cement (OPC), superplasticizer (SP), water, and coarse aggregate (CA). The model targets concrete compressive strength (MPa). Various configurations of experimental variables were taken into consideration. Supplementary Table S1 presents the results of the various experimental circumstances. Table 1 presents a descriptive statistical analysis of the laboratory-generated dataset to provide information. A variety of standard deviations is shown by the data in Table 1, which in turn aids in the construction of more comprehensive models. The input and target variables’ histogram are displayed in Figure 1. Using Pearson’s linear correlation, we also examine the linear correlation coefficients and their significance levels among the data factors [42]. Figure 2 shows several variable correlations. Linear correlation analysis is often used to discover input variables for modeling the final output, especially if numerous variables are unrelated to the target. Our experiment produced correlation coefficients for the five input variables and targets. The multicollinearity problem occurs when independent variables are highly correlated, resulting in incorrect machine learning model interpretation results [43]. The parameters due to their sensitivity, model parameters can cause large discrepancies between observed and predicted results [44]. Thus, predicted parameters must be precise to predict simulated values. Discovering sensitive factors is a crucial challenge for any statistical investigation. Evaluate sensitively Models created using various technologies were evaluated using statistical markers including R^2^, R, RMSE, and MAE [45]. The validation and testing of models differ slightly, but new machine learning techniques must estimate them to fully comprehend them. The model’s performance and dependability were assessed using four important parameters [46]. The various performance ratings and their corresponding ranks, which are generally used for purposes of assessment [47]. The following relationship Equation (5) shows how input variables affect procedure outcomes:

(1) $$R=\sqrt{1-[\frac{\sum_{i=1}^{N}{\left(O_{i}-I_{i}\right)}^{2}}{\sum_{i=1}^{N}{\left(O_{i}-\bar{P}\right)}^{2}}}]$$

(2) $$R^{2}=1-[\frac{\sum_{i=1}^{N}{\left(O_{i}-P_{i}\right)}^{2}}{\sum_{i=1}^{N}{\left(O_{i}-\bar{P}\right)}^{2}}]$$

(3) $$RMSE=\sqrt{\sum_{i=1}^{N}\left(\frac{{\left(O_{i}-P_{i}\right)}^{2}}{N}\right)}$$

The equation represents the relationship between the observed value (Oi), the expected value (Pi), the total number of observed samples (N), and the average of the forecasted value (Pi).

(4) $$SA=\frac{f_{max}\left(x_{i}\right)-f_{min}\left(x_{i}\right)}{\sum_{i}^{n}\left(f_{max}\left(x_{i}\right)-f_{min}\left(x_{i}\right)\right)}*100$$

where f_max_ (Xi) represents the highest estimated value, f_min_ (Xi) represents the lowest, and SA represents the sensitivity analysis.

## 4. Methodology

Parametric research on crucial parameters; ANN, RSM, and ANFIS architecture design; and dataset selection are all steps in the process of this study [23,48,49,50,51]. The prediction model was constructed by establishing input-target variable correlations using artificial neural fitting in MATLAB [52], central composite design in Design Expert [32], and a neuro-fuzzy inference system (ANFIS) [53]. Utilization of datasets from the literature ensured model reliability. The performance of the prediction model was evaluated using R, R^2^, MSE, and RMSE [54]. A wide range of precision of models and forecasting capacity measures were applied. A comprehensive parametric study examined input parameters and concrete compressive strength prediction.

### 4.1. Details of Response Surface Methodology (RSM)

**Figure 3:**
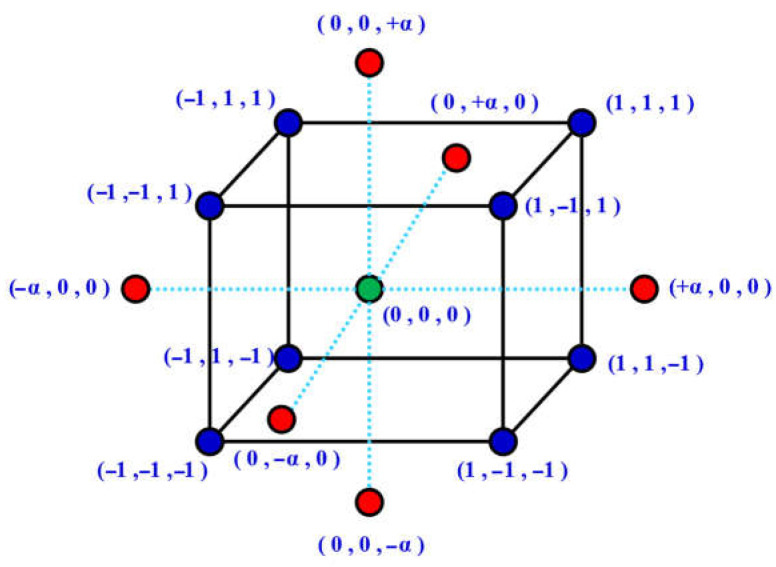
Schematic representation of factorial, axial, and center points in CCD [32].

The data (324 samples) was divided into 6 groups to facilitate data entry into Design Expert. Central composite design (CCD) was used to developed the groups [49,55]. Using three-dimensional and contour forms, this method shows the influence of three input factors on the response. Predicting any result within the variable’s changing range is made easier by these forms [56]. This method involves first identifying the essential variables, after which the implications of the variable are modeled and analyzed using statistical and mathematical tools [57]. Each numerical element is systematically manipulated over three (3) different levels. For axial/star points, they are as follows: +1 for high level, −1 for low level, +/−Alpha (+α), minus Alpha (−α), and the center point (mid-level) as shown in Figure 3.

The cement content, fine aggregate, and coarse aggregate are the three parameters—also referred to as independent variables—that are taken into account in the design. In coding terms, A, B, and C are independent variables, and R1–R6 are dependent variables. The variable was coded using Equation (5).

(5) $$Value\;in\;code=\frac{r-c}{h-c}$$

The experiment design specifies that r, c, and h denote the run’s number, factor center point, and factor highest value. Table 2 presents the factors and their respective ranges of variation.

### 4.2. Details of Artificial Neural Network (ANN)

**Figure 4:**
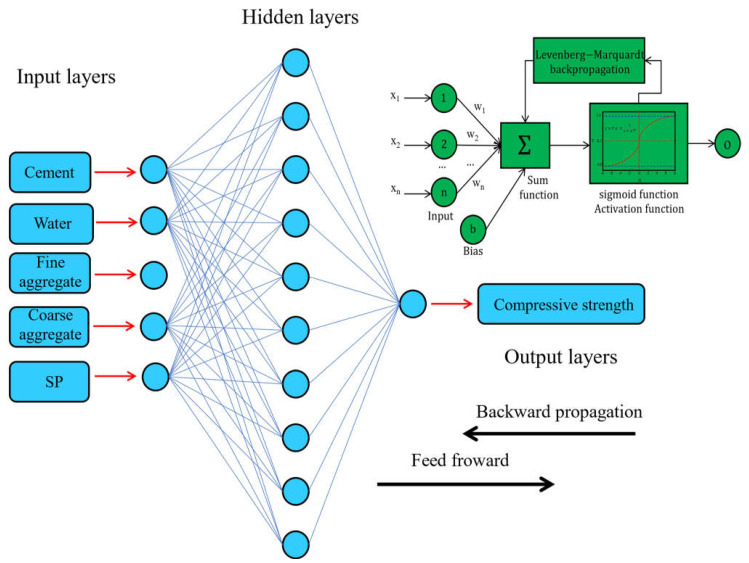
Schematic diagram of ANN models [60].

A computational model that mimics the neural system’s functional properties in the human brain through mathematical and numerical techniques is called an artificial neural network (ANN) [58]. The propagation learning process was carried out using the Levenberg–Marquardt approach with the MLP (multi-layer perceptron) feed-forward artificial neural network [59]. Output layers output signals, whereas input layers take input variables. This is the basic neural network architecture. In Figure 4, the input layer has five neurons representing the ANN with three independent variables, the hidden layer is the second, and the output layer is the ANN response.

Hidden layers are sometimes defined as the layers that sit between the input and output layers. Selecting a hidden layer is important because too many hidden layers cause the model to overfit, while too few hidden layers cause the model to underfit [61]. Table 1 describes the statistical features of the data. The input data were divided into three random categories: 70% for training samples (226 samples), 15% for validation (49 samples), and 15% for testing (49 samples). To avoid ANN low-learning-rate issues, we normalized the subject parameter values between suitable upper and lower limits. Equations (6) and (7) standardize min–max normalization for [−1, 1] upper and lower limit values [62].

(6) $$Data\;Standardization=\frac{x-\mu }{\sigma }$$

(7) $$Normalization\;data=\frac{Feature-{Feature}_{min}}{\;{Feature}_{max}-{Feature}_{min}}$$

### 4.3. Details of Neuro-Fuzzy Inference System (ANFIS)

**Figure 5:**
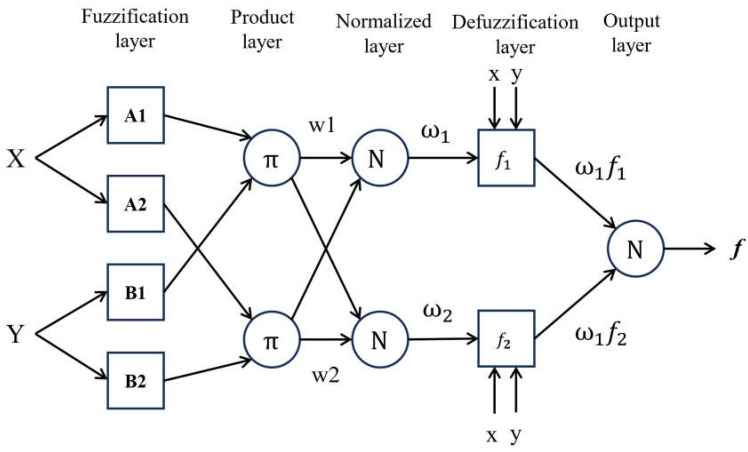
ANFIS architecture [63].

**Figure 6:**
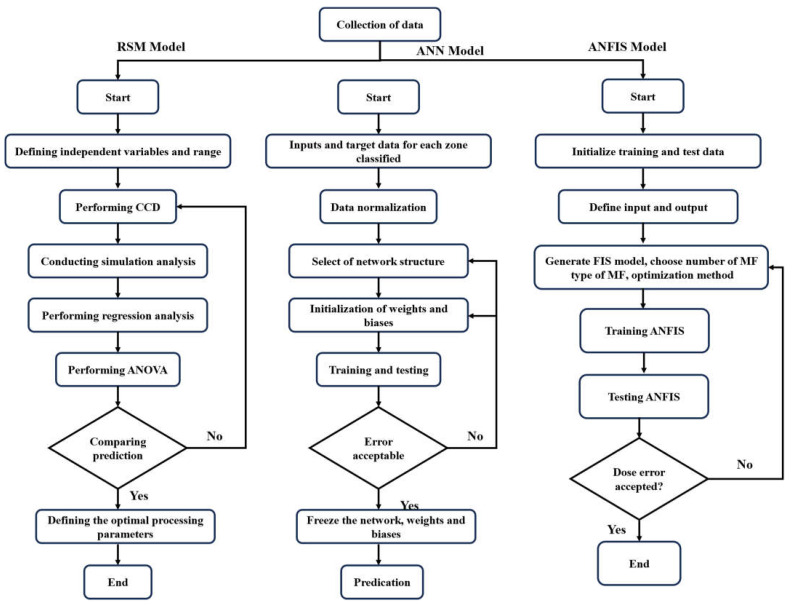
Flow chart of methodology.

The adaptive network framework of the adaptive neural network (ANFIS), a soft computing approach, combines ANN rules with fuzzy logic (FL) theories to construct a logical relationship between inputs and outputs [63]. The parameters are determined using an optimization technique during the adaptive neuro-fuzzy inference system (ANFIS) training process. Hybrid and backpropagation algorithms are the two main methods of optimization [64]. The gradient descent is the only method used in the backpropagation method to evaluate all parameters. The hybrid training method, on the other hand, suggests parameters by combining the gradient descent (GD) and least square estimation (LSE) techniques [65]. The five layers of the ANFIS process are the rule, fuzzification, normalization, defuzzification, and summation layers. Adaptive nodes, which might depict a square for variables that are changeable or a circle for variables that are not, are unique to each layer, as seen in Figure 5. The optimal ANFIS architecture was determined using a trial-and-error approach [66]. Figure 6 shows the flow chart of our methodology.

## 5. Results and Discussion

### 5.1. Artificial Neural Network (ANN)

**Figure 7:**
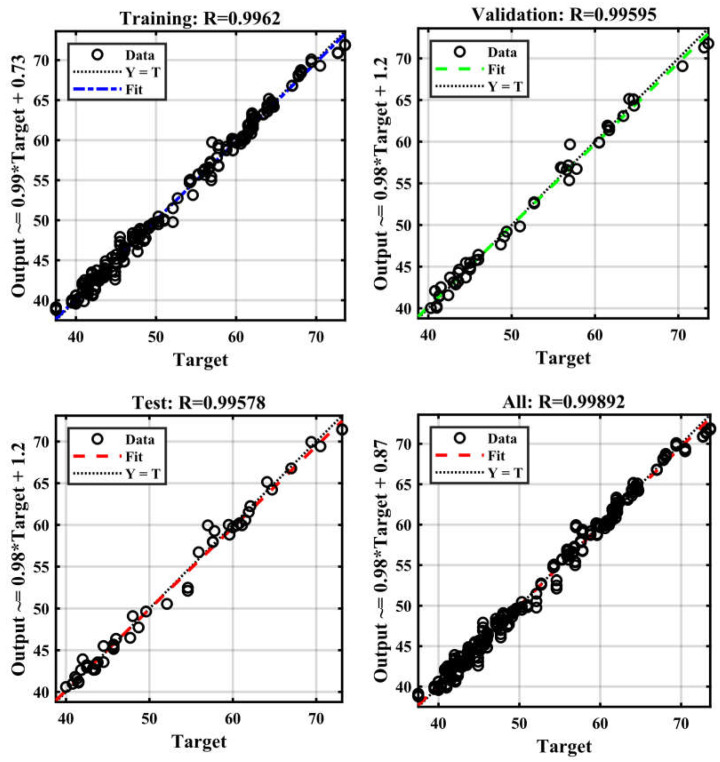
The coincidence between the output and target variables of training, validation, testing, and cumulative.

**Figure 8:**
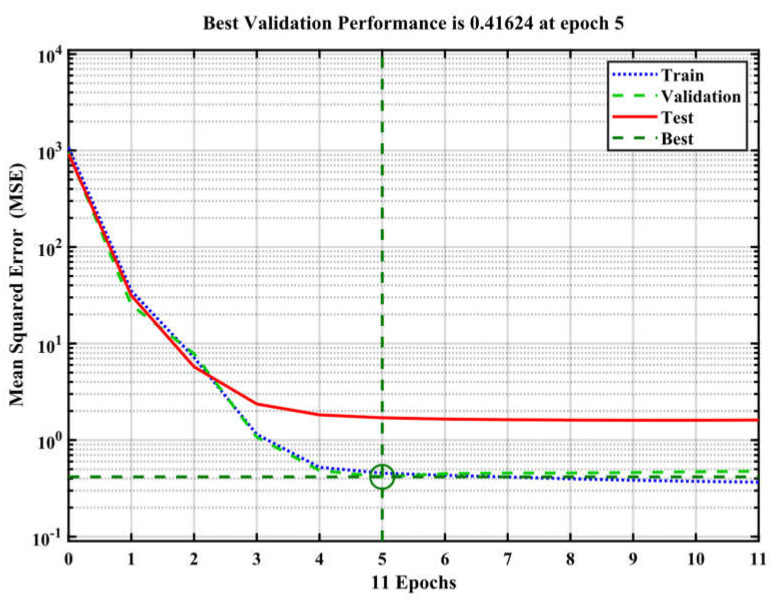
MSE of ANN.

**Figure 9:**
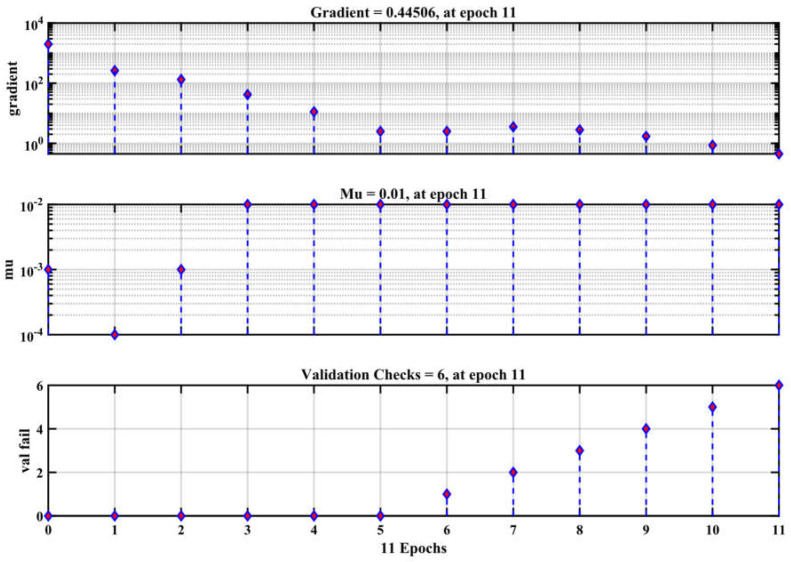
The training state of the ANN model.

This study involved the construction of an artificial neural network (ANN) to accurately forecast the compressive strength of conventional concrete [50]. The sample was divided into three parts for modeling: 70% was utilized for training, 15% for validation, and the remaining 15% for testing [60]. To mitigate the issue of overfitting, it was shown that enhancements in accuracy on the training dataset were in line with enhancements in accuracy on the validation dataset [67]. To assess the accuracy of the current models, the coefficient of determination (R^2^) was employed. Higher R^2^ and a reduced mean square error (MSE) indicate that the model forecasts the compressive strength of concrete with accuracy [68]. Based on the analysis using the ANN technique, Figure 6 displays the R correlation coefficient for training, validation, testing, and cumulative for compressive strength. As shown in Figure 7, the R correlation coefficients of training, validation, testing, and cumulative were 0.997, 0.996, 0.995, and 0.998. In every case where the R-value is higher than 0.9, a strong correlation between the actual and anticipated results is observed [69]. The input variables showed a nonlinear correlation with the model compressive strength prediction. The determination coefficient, R^2^, is 0.998. Figure 8 displays the network’s optimal validation performance and compressive strength MSE. As demonstrated in Figure 8, it was found that the MSE for the tested scenario in the training and validation datasets saturates with increasing epochs [62]. The 11th iteration of the training process marked the end of the process; however, it is important to remember that at this time, the error was higher than it had been in the fifth iteration for both the validation and testing data. According to this finding, the model may have begun to overfit the training set at iteration 5, as its performance did not improve beyond that point. This means the best time to discontinue using this model is after the fifth iteration. The model reached a minimal MSE of 0.416 at iteration 5. Lower MSE values, when the MSE value is 0, indicate superior performance and a lack of error. Figure 9 displays the ANN model’s training state and details the test termination at epoch 11 and the six-time error repeat after epoch 0. The weights from the first epoch, 0, are taken as the final weights and are considered as the reference point. Since the errors are repeated six times before the procedure is terminated, the validation check is equal to 6. MSE values and R for the compressive strength are shown in Table 3.

**Figure 10:**
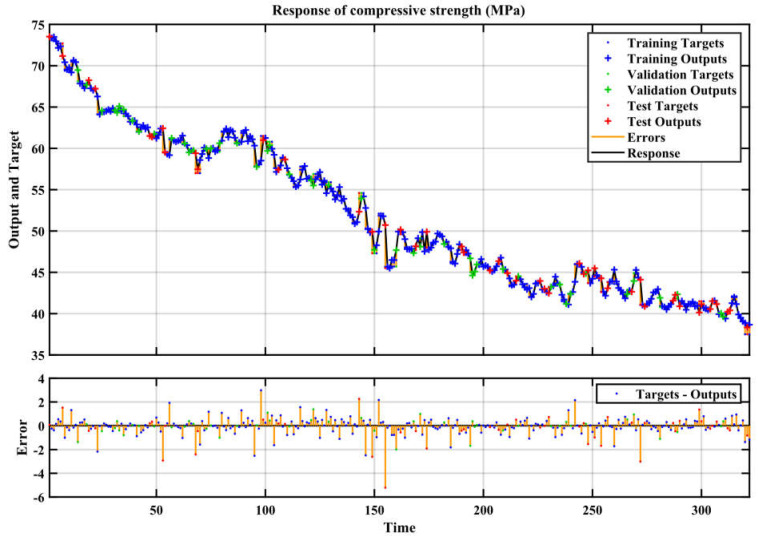
Response of output model 1 for time series for compressive strength.

The essential components of Figure 10 are training targets, training outputs, validation targets, validation outputs, test targets, test outputs, errors, and responds. The ability of MATLAB models to understand the link between the input and output parameters and to generalize is demonstrated by the tight match between the output values and target values, as shown in Figure 9. As shown in Figure 10, it is clear that the predicted results are very close to the laboratory results.

**Figure 11:**
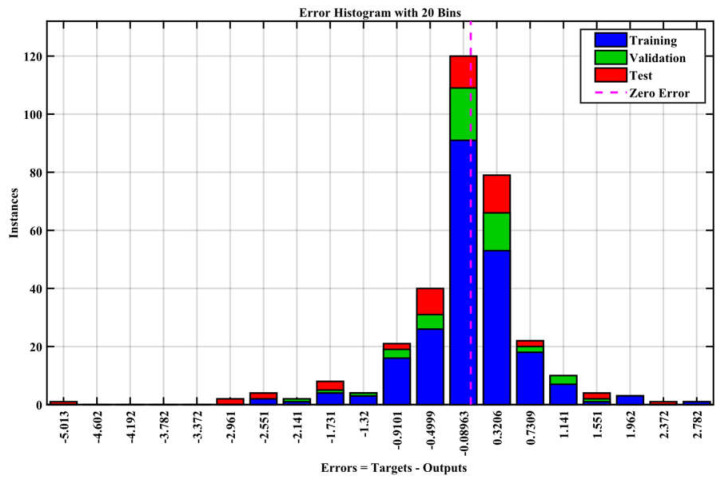
Error histogram.

**Figure 12:**
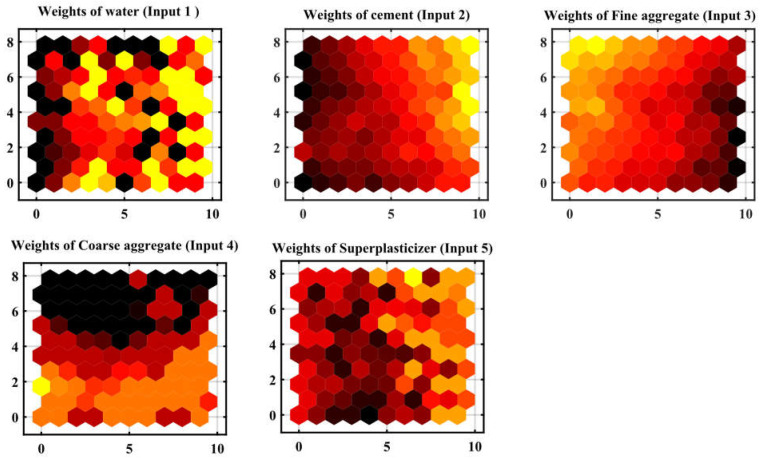
Component planes of the SOM for the 5 input variables.

Figure 11 shows error bin distribution to show the difference between actual and expected values. The substantial fraction of the dataset in smaller error bins implies the model’s concrete compressive strength estimates are correct. Based on a comparison of weight vector values for each variable in each map unit, the component planes of the five input variables, as shown in Figure 12, provided excellent assistance in interpreting the clusters obtained. Figure 12 showed low concentrations of water and superplasticizer. However, the main characteristic of these samples showed the highest levels of cement and fine and coarse aggregate. The effectiveness of the ANN model was tested using experimental results. Comparisons included sample size, input, output, and R^2^ value [70]. Table 4 shows that most researchers utilized similar input factors in this investigation. Data availability and variable significance have led some researchers to employ fewer input variables. Table 4 shows R^2^ values from 0.67 to 0.994 from the literature. In this analysis, the ANN model had a good R^2^ value of 0.998, near to the literature values. Compared to earlier models, this shows that the designed model operates at a high degree of accuracy. One difference between this study and others is sample size.

### 5.2. Response Surface Methodology (RSM)

**Figure 13:**
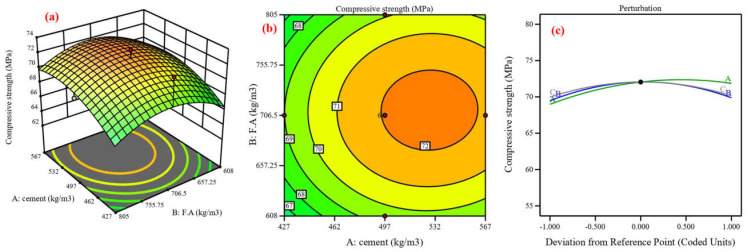
Compressive strength for response 1: (**a**) 3D view, (**b**) contour graph, and (**c**) perturbation plot.

**Figure 14:**
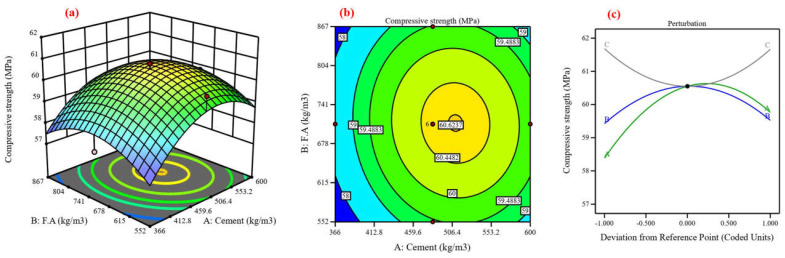
Compressive strength for response 2; (**a**) 3D view, (**b**) Contour graph and (**c**) perturbation plot.

**Figure 15:**
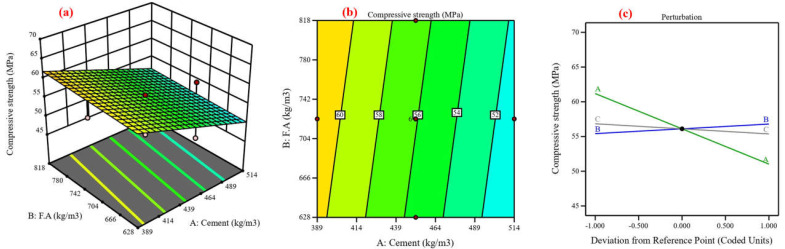
Compressive strength for response 3: (**a**) 3D view, (**b**) contour graph, and (**c**) perturbation plot.

**Figure 16:**
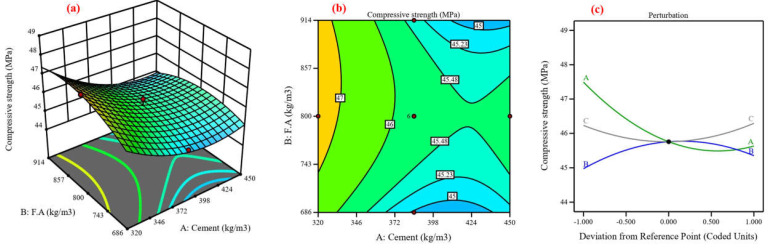
Compressive strength for response 4: (**a**) 3D view, (**b**) contour graph, and (**c**) perturbation plot.

**Figure 17:**
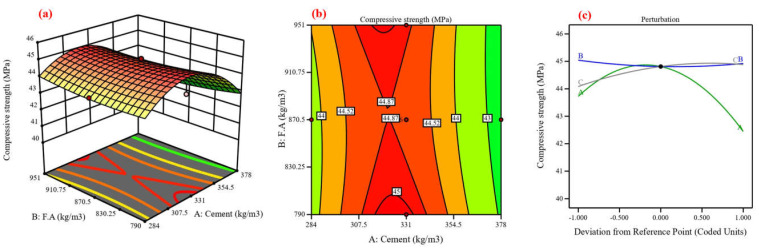
Compressive strength for response 5: (**a**) 3D view, (**b**) contour graph, and (**c**) perturbation plot.

**Figure 18:**
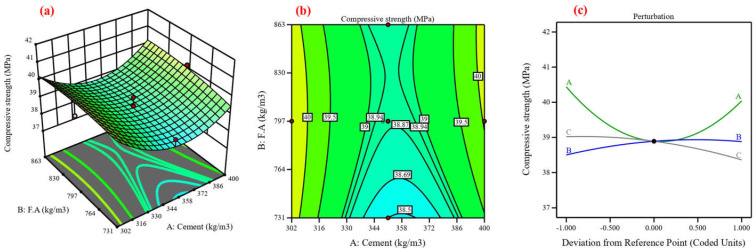
Compressive strength for response 6: (**a**) 3D view, (**b**) contour graph, and (**c**) perturbation plot.

Concrete’s mechanical and durability characteristics were predicted, and the process variables’ impact was assessed using central composite design (CCD) [48]. Using cement, fine aggregate, and coarse aggregate as variables, the CCD model was utilized in this study to analyze six responses in compressive strength [82]. Twenty experimental points, or runs, are proposed for each of the three numerical factors: six factorial points without replication, eight axial points without replication, and one center point with eight replications, resulting in ten runs [83]. The lack-of-fit analysis, coefficient of determination, and F-value are components of an ANOVA [84]. The coefficient of determination (R^2^), which possesses three properties—anticipated, adjusted, and R^2^—expresses the extent of variation between the experimental and predicted values [37]. Figure 13, Figure 14, Figure 15, Figure 16, Figure 17 and Figure 18 show the compressive strength from responses 1 to 6 in several types, including a 3D view, contour graph, and perturbation. Table 5 shows the ANOVA results for the parameters of the CS of HSC. Compressive strength was mathematically predicted from responses 1 through 6 using the equations derived from Equations (8)–(13).

C.S for response 1 = 72.05 + 1.44 C + 0.21 F.A + 0.09 C.A + 0.087 C ∗ F.A + 1.09 C ∗ C.A + 0.912 F.A ∗ C.A − 1.63C^2^ − 2.38 F.A^2^ − 1.98 C.A^2^ (8)

C.S for response 2 = 60.55 + 0.69 C + 0.05 F.A − 0.01 C.A − 0.112 C ∗ F.A + 0.137 C ∗ C.A − 0.1375 F.A ∗ C.A − 1.48C^2^ − 1.08 F.A^2^ + 1.12 C.A^2^ (9)

C.S for response 3 = 56.10 − 5.08 C + 0.69 F.A − 0.73 C.A(10)

C.S for response 4 = 45.76 − 0.93 C + 0.19 F.A + 0.03 C.A − 0.3 C ∗ F.A − 0.125 C ∗ C.A − 0.275 F.A ∗ C.A + 0.08045 C^2^ − 0.595 F.A^2^ + 0.504 C.A^2^ (11)

C.S for response 5 = 44.81 − 0.64 C − 0.06 F.A + 0.4 C.A − 0.2 C ∗ F.A + 0.775 C ∗ C.A − 0.325 F.A ∗ C.A + 1.73 C^2^ + 0.172 F.A^2^ − 0.327 C.A^2^ (12)

C.S for response 6 = 38.89 − 0.2 C − 0.19 F.A − 0.33 C.A − 0.187 C ∗ F.A + 0.363 C ∗ C.A + 0.6875 F.A ∗ C.A + 1.35C^2^ − 0.2 F.A^2^ − 0.2 C.A^2^ (13)

For given levels of each element, the coded equation can predict the reaction. High factor levels are +1 and low levels are −1 by default. Comparing factor coefficients with the coded equation assists in assessing variable influence [85]. *p*-values of responses 1 to 6 were 0.028, 0.01, 0.003, 0.023, 0.002, and 0.026 are less than 0.05, indicating that the model is highly significant [86].

The model F-value for six responses was 1.48, 4.92, 6.96, 3.88, 7.94, and 3.37, which implies the model is not significant relative to the noise. As shown in Figure 13, Figure 14, Figure 16, Figure 17, and Figure 18a, the process order was quadratic while the process order in Figure 15a was linear. As shown in Figure 13, Figure 14, Figure 15, Figure 16, Figure 17 and Figure 18b, the maximum predicted compressive strength for six responses was 73.6, 61.4, 65.2, 48.1, 45, and 41.3 MPa, while the minimum predicted compressive strength was 62.1, 57.6, 45.9, 44.2, 40.6, and 37.6 MP. As shown in Figure 13a,b the predicted compressive strength increased by 2.56%, 3.96%, 4.21%, and 3.13% for 608 kg/m^3^ of fine aggregate while the increase in compressive strength was 2.36%, 3.94%, 4.27%, and 3.30% for 706.5 kg/m^3^ at cement content 462, 497, 532, and 567 kg/m^3^, respectively. This increase in compressive strength is a result of the high cement content contributing to the formation of dense C-S-H, which provides a dense microstructure [87]. RSM perturbation plots highlighted significant parameters by showing variations in factor response when each factor moved from the reference point, the zero-coded level of each factor, with all other factors held constant at the reference value [88]. The compressive strength (CS) perturbation plot in Figure 13c revealed that cement, fine aggregate, and coarse aggregate have a similar effect on the compressive strength. It was also revealed that the compressive strength decreases slightly with the increase in the proportion of fine and coarse aggregate. As shown in Figure 14a,b the predicted compressive strength was 59.56, 60.26, 60.61, 60.56, 60.13, and 59.60 MPa at cement content 506.4 kg/m^3^ for fine aggregate 552, 615, 678, 741, 804, and 867 kg/m^3^. Reducing water content also contributed to the effect on compressive strength with a positive impact [88]. According to the perturbation plot in Figure 14c, cement (A) and fine aggregate (B) have the highest compressive strength (CS) near the reference point (middle region), while coarse aggregate decreases compressive strength. As shown in Figure 15a,b, the predicted compressive strength decreased by 2.94%, 6.77%, 9.95%, 13.16%, and 16.41% for 704 kg/m^3^ of fine aggregate at cement content 414, 439, 464, 489, and 514 kg/m^3^, respectively. The perturbation plot in Figure 15c revealed that the increase in cement content led to decreased compressive strength. At the same time, the influence of fine aggregate (B) and coarse aggregate (c) on compressive strength was observed to be negligible. The modeling of responses 4 and 6 revealed a correspondence in the behavior of the variables in terms of compressive strength, as shown in Figure 16 and Figure 18a,b and perturbation plot Figure 16 and Figure 18c, which is due to the convergence of the variables’ values. As shown in Figure 17a,b the predicted compressive strength was 43.78, 44.68, 44.84, 44.06, and 42.53 MPa at fine aggregate 870.5 kg/m^3^ for cement content 284, 307.5, 331, 354.5, and 378 kg/m^3^. In Figure 17c, the perturbation plot demonstrated that the amount of cement enhanced compressive strength. Meanwhile, fine aggregate (B) and coarse aggregate (c) had little effect on compressive strength. Table 6 illustrates the efficiency of the RSM model in prediction by comparing its development with previous studies.

### 5.3. Neuro-Fuzzy Inference System (ANFIS)

**Figure 19:**
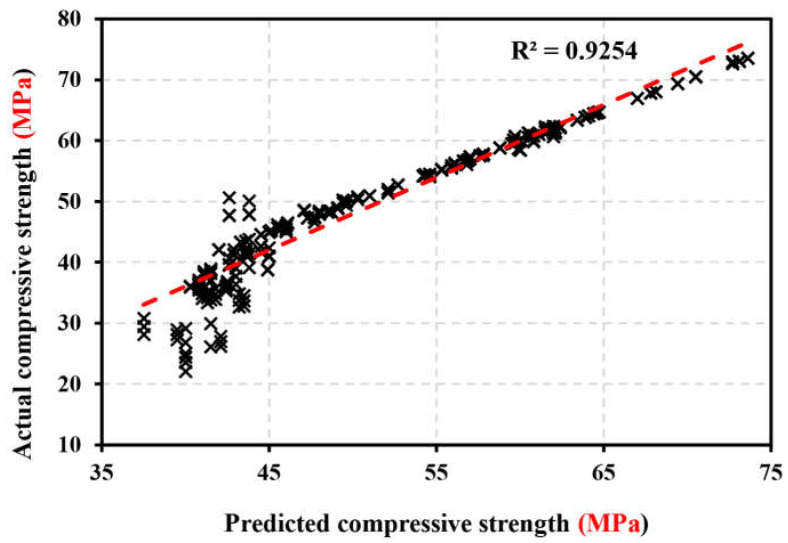
The predicted and experimental values of compressive strength.

The fuzzy inference system in the current work is created using the subtraction clustering technique [98]. The type and quantity of membership functions are chosen solely by the hybrid learning strategy, which employs gradient descent and the least-squares method to find a workable set of antecedent and subsequent parameters [99]. The ANFIS model in the current prediction study was developed using the quick learning technique, in conjunction with the Fuzzy Logic Toolbox and MATLAB [26]. The optimum ANFIS architecture was determined using a trial-and-error approach. To minimize inaccuracy, 16 epochs were used during training. The model’s training results reveal the lowest error after 3 epochs and probable convergence after 16 epochs. Figure 19 shows the expected and experimental values of compressive strength. The 324 testing data points have a 0.925 percent correlation. Training error size-wise, expected, and experimental values are near. The research shows that most test results match expected values.

### 5.4. Analysis of ANN and ANFIS Model Sensitivity

**Figure 20:**
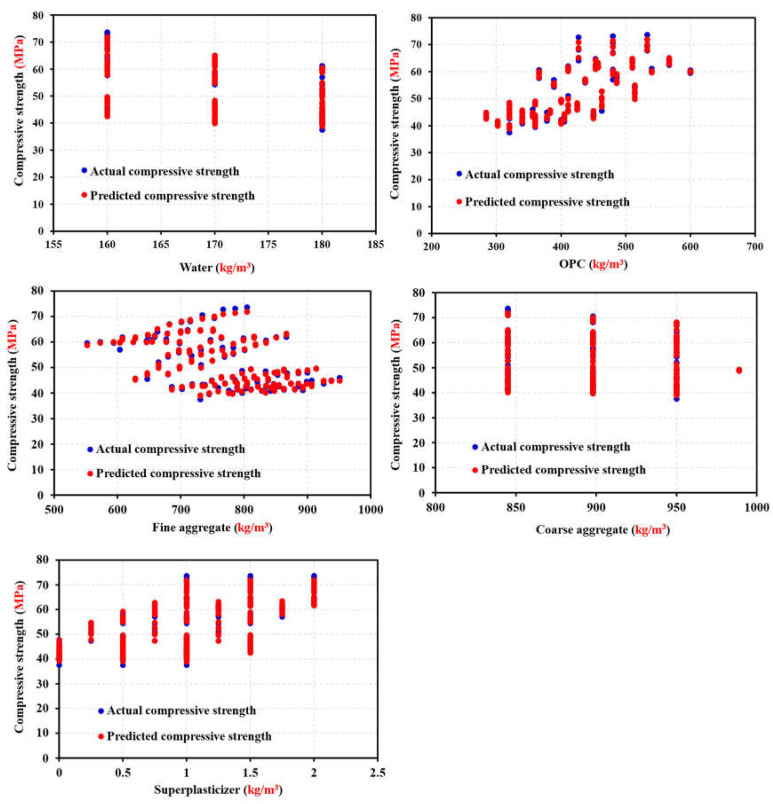
Model parameter of sensitivity analysis.

**Figure 21:**
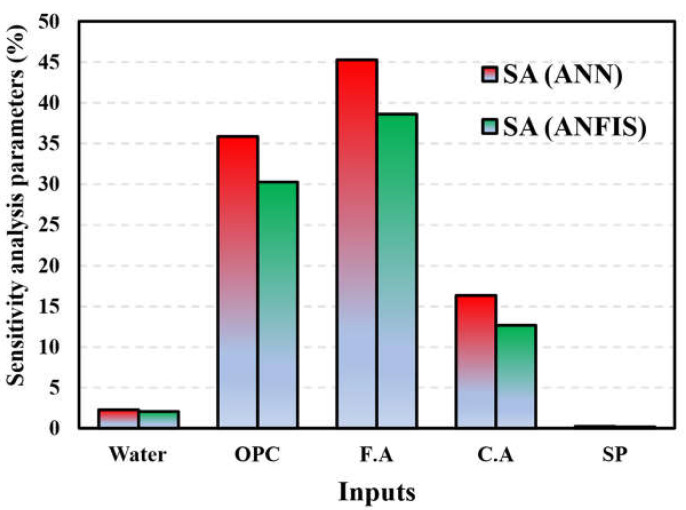
Sensitivity analysis of the input parameters.

The impact of the input variables on the output variables of the dataset was evaluated using sensitivity analysis (SA). Quantifying the relationship between uncertainty in a model’s inputs and outputs can be achieved using several techniques provided by SA [75]. The SA normally evaluates how sensitive the model is to parameter and data uncertainty. Input variables affect output variables more with higher SA values [100]. SA linked ML model outcomes to input variable count and dataset. As illustrated in Figure 20, the correlation coefficients of all input variable combinations were determined to eliminate multicollinearity. As the cement content and fine aggregate are increased, the concrete compression strength increases, as shown in Figure 20, and OPC showed a greater correlation coefficient than the other variables. The correlation coefficients of CA, water, OPC, FA, and SP were −0.31, −0.72, −0.45, −0.093, and −0.59, respectively. These parameters significantly decreased the compressive strength of the concrete, with cement being the most significant factor. The models utilize two scenarios since the ANN, RSM, and ANFIS models will include/exclude various independent variables because of the significant correlation between “fine aggregate” and “coarse aggregate” [92]. Figure 21 illustrates how input parameters affect the estimate of the ANN’s compression strength. sensitivity analysis revealed that the OPC, which accounted for 45.29% of the total impact, was the most important component [100]. The fine aggregate came in second at 35.87%, and the coarse aggregate at 15.35%. These results agree with the findings of previous research [93].

**Figure 22:**
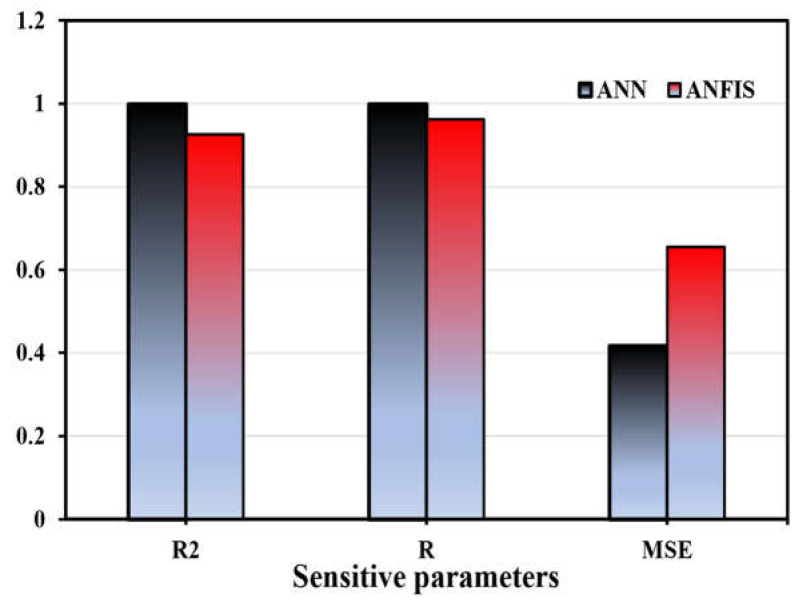
Sensitive parameters.

The influence of additional input parameters on the calculation of concrete’s compressive strength using an artificial neural network (ANN) was shown to be diminished. Concerning the SA results, the following aspects can be concluded. Firstly, most of the variables related to cement and aggregate show a stronger influence on the compressive strength of concrete than that of the variables related to water +SP, as shown in Figure 22. A value of 1 for the R^2^ coefficient represents the optimal level of fit. The statistical model’s prediction accuracy can be measured using equation for R [44]. A value of R or R2 nearer to 1 implies that the projected value is closer to the experimental value [94].

### 5.5. Validation and Comparison of RSM, ANN, and ANFIS Models

**Figure 23:**
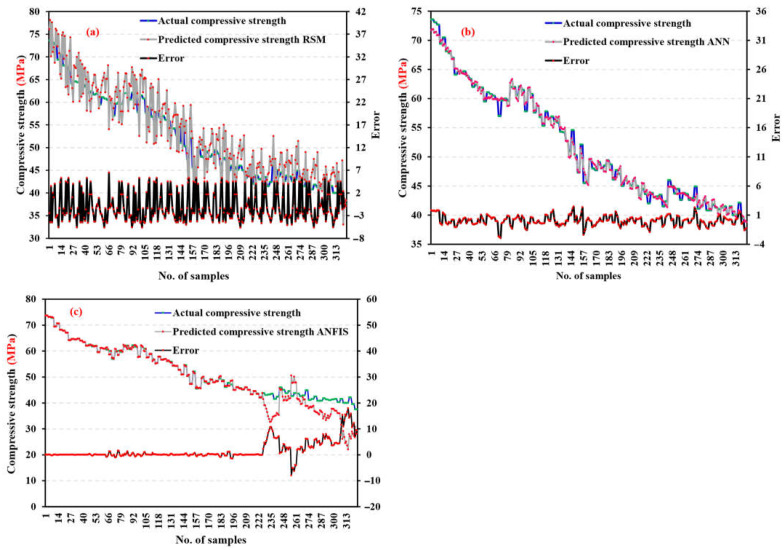
Comparing experimental compressive strength to predicted compressive strength using (**a**) RSM, (**b**) ANN, and (**c**) ANFIS.

In recent years, RSM-, ANFIS-, and ANN-based degrees of experimentation have become the most widely used models and process improvement methodologies [79,101]. The predicted data and the mean actual data, shown in Figure 23, were compared to assess the correctness of the created ANN, ANFIS, and RSM models. The correlation between the actual and predicted values was examined to determine how accurate the mathematical models performed. These results demonstrated a significant correlation, which confirmed that the mathematical models correctly anticipated the outcomes [102]. RSM was marginally less accurate than ANN models in predicting responses [50]. To validate the RSM, ANN, and ANFIS models, the determination coefficient (R^2^) was used to compare actual and anticipated results. As shown in Figure 23a, it is clear that the actual results and the results predicted by ANN are close, but in Figure 23b, it is clear that there is a divergence between the actual and predicted results. This proves the accuracy of the ANN model in predicting the RSM model [50]. From Figure 23a–c, the maximum difference between the actual and predicted results was 2.95, 6.5, and 17.9 MPa, while the minimum difference between the actual and predicted results was 2.48, 5.65, and 8.05 MPa by the ANN, RSM, and ANFIS models, respectively. Using a typical reference dataset, the suggested ANN and ANFIS models predict bond strength compared to other models in the literature [53]. Understanding how bond strength outputs change with a single variable when all others are fixed is helpful. Table 7 displays that the highest R-squared and lowest MSE belong to ANN and ANFIS. The performance evaluation of the produced artificial neural network (ANN), response surface methodology (RSM), and neuro-fuzzy inference system (ANFIS) models is presented statistically in Table A1.

## 6. Discussion

This study aims to improve the accuracy of forecasts for high-strength concrete by utilizing artificial neural network [43], response surface methodology [104], and neuro-fuzzy inference system [26] modeling methodologies. The precise organization of the experimental data facilitated the implementation of ANN, RSM, and ANFIS models. The quantity of datasets has a notable impact on the accuracy of a model [99]. This model comprises a total of 324 data points. The optimum predictor was identified by comparing the accuracy of artificial neural network (ANN), response surface methodology (RSM), and neuro-fuzzy inference system techniques [53,59]. Artificial neural network (ANN) model outperformed the response surface method (RSM) [78] and neuro-fuzzy inference system [26] models in terms of the R^2^ value, the discrepancy between actual and predicted results, and the accuracy of error estimates. Nevertheless, the results of the RSM model exhibited a high level of concordance with the experimental data [71]. Prior studies have shown that the artificial neural network (ANN) method outperforms other machine learning (ML) techniques in accurately predicting various characteristics [103]. The quantity of inputs and datasets required to run algorithms determines how effective a machine learning (ML) method is, which makes it challenging to identify and recommend the best ML strategy for outcome prediction across a range of scientific domains. Applications of ANN research can be helpful to the construction sector since they can expedite the development of quick and low-cost methods for assessing material properties [105]. From Table 4, it was shown that the ANN model that was developed in this research proved to have a higher prediction efficiency than those in previous research. The high efficiency in prediction is due to many reasons, including that the large number of ANN inputs improves the ability of prediction as it reduces errors in operations [106]. Some research has shown that one of the reasons for increasing the ability to predict results is introducing variables by normalizing parameter values to avoid problems with the low learning rate of ANN [60]. Representation learning, nonlinear modeling, scalability, adaptability, and advances in regularization and ensemble learning enable machine learning (ML) such as artificial neural networks (ANNs) and neuro-fuzzy inference systems (ANFISs) to predict outcomes in a robust way in many applications [12,43]. As shown in Table 4, it turned out that the ANN model in this study achieved the highest prediction rate with an R^2^ of 0.998 compared to the previous literature [54,107,108]. As can be seen from Table 6, the RSM model constructed for this study outperformed previous research with regard to prediction efficiency. This study achieved a prediction rate R^2^ of 0.963, which is a high percentage compared to the previous literature [45,59,84]. One of the important reasons for improving the prediction accuracy of the RSM model is choosing the input variables correctly and doing correct modeling [109]. Table 7 shows that the ANFIS model in this study had the greatest prediction rate, with an R^2^ of 0.926 compared to the previous literature [53,97,101,109]. Different statistical indicators were used to compare the results in pervious study [110]. Table 8 explains the results extracted from the artificial neural network (ANN) and neuro-fuzzy inference system.

## 7. Limitations and Future Studies

The RSM approach requires additional experimental data for points linked to −1, +1, 0, −α, and +α to accomplish high-accuracy data prediction. To assess the impact of various variables on prediction, the ANN must gather additional data incorporating a wider range of variables. Future work can be summarized as follows:

- Make a comparison between the outputs based on the amount of data entered into the program.
- Using experimental data for prediction via genetic programming (GEP), multi-layer perceptron neural networks (MLPANNs), and GANNs to forecast the properties of concrete.
- Most of the researches focused on predicting the hardening properties of concrete, so we recommend conducting researches to predict the durability and microstructure of concretes.
- The resulting model’s prediction reliability should be tested by comparing it to the new mixture’s compressive strength.

## 8. Conclusions

This article utilized an artificial neural network (ANN) and neuro-fuzzy inference system (ANFIS) model to forecast the compressive strength of high-strength concrete (HSC). The predicted results were then contrasted with a response surface method (RSM) model. The purpose was to compare the machine learning and non-machine learning approaches to under the differences and behavior and to identify the best approach for predicting the compressive strength of construction material. Key findings are summarized below:

- ANN and ANFIS models can manage large databases with many important factors and change nonlinearity with their robust computational approaches.
- Based on R^2^ and variance between real and predicted results, the ANN model predicted the compressive strength of HSC more accurately than the RSM model.
- Since R^2^ exceeds 0.99 in training and testing, the ANN model can capture the complex nonlinear connection between the five input parameters and HSC compressive strength.
- Instead of the RSM model, the ANN model is recommended for HSC strength prediction due to its greater prediction capacity. The ANN model can estimate HSC compressive strength before laboratory compression experiments, reducing time and cost.
- Testing data were used to validate the ANFIS model after it was built using training data. The RMSE was calculated to be 0.655, and the correlation coefficient was estimated to be 0.925.
- Of the five input variables for ANN and ANFIS, cement and fine aggregate are the most important and sensitive to compressive strength.
- According to the ANN model’s sensitivity analysis, cement (45.29%) is the main variable affecting compressive strength. As another major variable in compressive strength prediction, fine aggregate contributes 35.87%. However, superplasticizer (0.227%) had the lowest incidence. The compressive strength of HSC with ANN increases more with cement and fine aggregate, whereas superplasticizer decreases it.
- ANOVA results validated the statistical significance of including all model parameters due to the extraordinarily low *p*-value.
- The three most influential factors in HSC compressive strength prediction are cement, fine aggregate, and coarse aggregate, with R^2^ 0.963.
- ANN, RSM, and ANFIS predicted a maximum compressive strength of 71.91, 78.1, and 73.61 MPa, and a minimum compressive strength of 38.8, 33, and 22.07 MPa, respectively.
- The proposed ANN model can be utilized to reduce the experimental specimens, required to determine the compressive strength of HSC

## Figures and Tables

**Table 1:** Features of descriptive statistics for modeling-relevant variables.

	Water (kg/m^3^)	OPC (kg/m^3^)	F.A (kg/m^3^)	C.A (kg/m^3^)	SP (kg/m^3^)	CS (MPa)
Mean	170	417.81	767.71	898.51	0.95	51.93
Median	170	411	769.5	898	1	48.9
SD	8.18	77.03	85.45	43.82	0.55	9.45
Minimum	160	284	552	845	0	37.5
Maximum	180	600	951	989	2	73.6
Type	Input	Input	Input	Input	Input	Output

SD: standard deviation; CS: compressive strength.

**Table 2:** Factors and factor levels for RSM.

	Response	Factor	Code	Factors Level of Code		
				Low Level −1	Intermediate Level 0	High Level +1
Compressive strength	Response 1	Cement (kg/m^3^)	A	427	497	567
		F.A (kg/m^3^)	B	608	706.5	805
		C.A (kg/m^3^)	C	845	897.5	950
	Response 2	Cement (kg/m^3^)	A	366	483	600
		F.A (kg/m^3^)	B	522	694.5	867
		C.A (kg/m^3^)	C	845	897.5	950
	Response 3	Cement (kg/m^3^)	A	389	451.5	514
		F.A (kg/m^3^)	B	628	723	818
		C.A (kg/m^3^)	C	845	897.5	950
	Response 4	Cement (kg/m^3^)	A	320	385	450
		F.A (kg/m^3^)	B	686	800	914
		C.A (kg/m^3^)	C	845	917	989
	Response 5	Cement (kg/m^3^)	A	284	331	378
		F.A (kg/m^3^)	B	790	870.5	951
		C.A (kg/m^3^)	C	845	897.5	950
	Response 6	Cement (kg/m^3^)	A	302	351	400
		F.A (kg/m^3^)	B	731	797	863
		C.A (kg/m^3^)	C	845	897.5	950

**Table 3:** R and MSE for training, validation, testing, and cumulative.

	R	MSE
Training	0.996	0.987
Validation	0.995	0.417
Testing	0.995	0.457
All	0.998	---

**Table 4:** Comparing the prediction of compressive strength of concrete with the current research.

Machine Learning Algorithm	NO. Sample	Variable Concrete Content	R^2^-Value	Ref.
ANN	150	Waste marble powder, cement, superplasticizer, silica fume, fly ash, water.	0.976	[60]
ANN	239	Cement, coarse, fine aggregate, and w/c	0.85	[71]
ANN	300	Cement, fly ash, coarse, fine aggregate, superplasticizer, and w/c	0.933	[72]
ANN	103	External diameter of CFST composite column filled with recycled concrete, the thickness of the steel tube, length of specimen, the proportion of replaced recycled coarse aggregates, compressive strength of recycled concrete, and yield stress of the steel tube	0.988	[73]
ANN	13	W/c, cement, water, coarse, fine aggregate, and condensed milk can (tin) fibers	0.982	[74]
ANN	40	Beam dimensions, compressive strength of SCGC under ambient and marine exposure conditions, time of exposure, the tensile strength of the BFRP bar, tensile strength of steel reinforcement bar, and shear span–depth ratio	0.958	[75]
ANN	50	Cement, water, sand, aggregate, w/b, and eggshell powder	0.825	[76]
ANN	17	Cement, w/c, coarse, fine aggregate, and foam volume	0.98	[77]
ANN	55	Cement, admixtures, water, coarse, fine aggregate, and superplasticizer	0.929	[78]
ANN	234	Testing age, OPC, sand, coarse aggregate, fine aggregate, w/c, SP/C, CRA/total coarse aggregate, and FRA/total fine aggregate	0.984	[79]
ANN	17	Cement, water, natural coarse aggregates, recycled coarse aggregates, and natural sand	0.994	[80]
ANN	60	Cement, admixtures, water, coarse, fine aggregate, and waste	0.67	[61]
ANN	220	Sand/cement ratio, dry density, and water/cement ratio	0.972	[81]
ANN	324	Cement, water, coarse, fine aggregate, and superplasticizer	0.998	The current study

**Table 5:** ANOVA results for the parameters for the compressive strength.

Response 1						Response 2					
Source	S.S	M.S	F-value	*p*-value		Source	S.S	M.S	F-value	*p*-value	
Model	169.21	18.00	1.48	0.028	Significant	Model	20.55	2.280	4.92	0.01	Significant
A-cement	20.74	20.740	1.63	0.231		A-Cement	4.76	4.76	10.27	0.00	
B-FA	0.441	0.441	0.035	0.856		B-FA	0.025	0.025	0.054	0.822	
C-CA	0.081	0.081	0.006	0.938		C-CA	0.001	0.001	0.002	0.964	
AB	0.061	0.061	0.005	0.946		AB	0.101	0.101	0.218	0.651	
AC	9.46	9.46	0.744	0.409		AC	0.151	0.151	0.326	0.581	
BC	6.66	6.66	0.524	0.486		BC	0.151	0.151	0.326	0.581	
A^2^	7.28	7.28	0.572	0.467		A^2^	6	6	12.940	0.005	
B^2^	15.54	15.54	1.22	0.295		B^2^	3.19	3.19	6.88	0.026	
C^2^	10.75	10.75	0.845	0.38		C^2^	3.47	3.470	7.47	0.022	
Residual	127.24	12.72	R^2^	0.986		Residual	4.64	0.464	R^2^	0.916	
Lack of Fit	127.24	25.45	Adj. R^2^	0.973		Lack of Fit	4.64	0.928	Adj. R^2^	0.901	
Cor Total	296.45		Pred. R^2^	0.963		Cor Total	25.190		Pred. R^2^	0.887	
Response 3						Response 4					
Source	S.S	M.S	F-value	*p*-value		Source	S.S	M.S	F-value	*p*-value	
Model	268.15	89.38	6.96	0.003	Significant	Model	14.51	1.61	3.880	0.023	Significant
A-Cement	258.06	258.06	20.1	0.001		A-Cement	8.65	8.65	20.81	0.001	
B-FA	4.76	4.76	0.371	0.551		B-FA	0.361	0.361	0.869	0.373	
C-CA	5.33	5.33	0.415	0.529		C-CA	0.009	0.009	0.022	0.886	
Residual	205.38	12.84				AB	0.72	0.72	1.73	0.218	
Lack of Fit	109.97	10	0.524	0.827		AC	0.125	0.125	0.301	0.595	
Pure Error	95.41	19.08		R^2^	0.956	BC	0.605	0.605	1.46	0.255	
Cor Total	473.53	Adj. R^2^	0.9431	Pred. R^2^	0.933	A^2^	1.78	1.78	4.28	0.065	
						B^2^	0.975	0.975	2.35	0.157	
						C^2^	0.7	0.7	1.68	0.224	
						Residual	4.16	0.416	R^2^	0.942	
						Lack of Fit	4.16	0.832	Adj. R^2^	0.924	
						Cor Total	18.67		Pred. R^2^	0.90	
Response 5						Response 6					
Model	28.570	3.17	7.940	0.002	Significant	Model	13.480	1.500	3.730	0.026	Significant
A-Cement	4.100	4.1	10.250	0.01		A-Cement	0.400	0.400	0.998	0.341	
B-FA	0.036	0.036	0.09	0.77		B-FA	0.361	0.361	0.900	0.365	
C-CA	1.600	1.6	4	0.073		C-CA	1.090	1.090	2.720	0.13	
AB	0.320	0.32	0.801	0.392		AB	0.281	0.281	0.702	0.422	
AC	4.810	4.81	12.03	0.006		AC	1.050	1.050	2.620	0.137	
BC	0.845	0.845	2.11	0.177		BC	3.780	3.780	9.430	0.012	
A^2^	8.200	8.2	20.53	0.001		A^2^	5.010	5.010	12.500	0.005	
B^2^	0.082	0.082	0.205	0.66		B^2^	0.110	0.110	0.274	0.612	
C^2^	0.295	0.295	0.737	0.411		C^2^	0.110	0.110	0.274	0.612	
Residual	4	0.4	R^2^	0.912		Residual	4.010	0.401	R^2^	0.902	
Lack of Fit	4	0.799	Adj. R^2^	0.876		Lack of Fit	3.8	0.760	Adj. R^2^	0.924	
Cor Total	32.56		Pred. R^2^	0.856		Cor Total	17.49		Pred. R^2^	0.9	

**Table 6:** Comparing concrete compressive strength predictions to existing research.

	Variable Concrete Content	R^2^-Value	Ref.
RSM	Cement, recycled concrete aggregates, and slump	0.95	[89]
RSM	Pulverized fuel ash (PFA), stone powder (SP), and silicon fume (SC)	0.85	[45]
RSM	Recycled concrete aggregates, silica fume, and ground-granulated blast-furnace slag	0.95	[90]
RSM	Crumb rubber and fly ash	0.95	[91]
RSM	Waste foundry sand and curing	0.90	[85]
RSM	Waste marble aggregate and stone dust	0.94	
RSM	Plastic, silica fume, and time	0.94	[92]
RSM	Crumb rubber (CR)–NaOH pretreatment, NaOH, and crumb rubber	0.97	[93]
RSM	Ground granulated blast furnace slag and nano silica	0.93	[94]
RSM	Content of SR, content of NS, and water binder ratio	0.93	[95]
RSM	Foam, waste marble powder, and rice husk ash	0.94	[96]
RSM	Heating temperatures, soaked time, and cooling methods	0.79	[97]
RSM	Cement, water, coarse, fine aggregate, and superplasticizer	0.963	The current study

**Table 7:** Comparison of validation data.

Model	ANN	ANFIS	[53]	[93]	[103]	[97]
R^2^	0.998	0.925	0.92	0.07	0.89	0.76
R	0.998	0.962	0.959	0.265	0.944	0.871
MSE	0.416	0.655	1.15	23.05	5.024	2.8

**Table 8:** Comparison between different methods of prediction.

	R	R^2^	MSE	RMSE	MAE	erMAX	EPR
ANN	0.9989	0.98	0.417	0.646	0.035	0.045	1.05
ANFIS	0.926	0.962	0.655	0.809	0.045	0.019	1.813

## Data Availability

The raw data of this article will be made available by reasonable request from the corresponding authors.

## Supplementary Materials

The following are available online at https://www.mdpi.com/article/10.3390/ma17184533/s1, Table S1. The experimental dataset.

## Appendix A

**Table A1:** Actual and predicted results of RSM and ANN for compressive strength.

ID	Actual C.S	ANN	RSM	ANFIS	ID	Actual C.S	ANN	RSM	ANFIS
1	73.6	71.78	76.1	73.60	51	61.7	61.98	66.02	61.70
2	73.6	71.86	78.1	73.60	52	61.9	60.83	63.226	61.75
3	73.6	71.92	70.1	73.60	53	61.9	61.32	62.4	61.76
4	73.1	71.30	77.6	73.08	54	61.9	61.53	62.8	61.90
5	73.1	71.41	71.1	73.08	55	59.5	59.87	58.6	59.50
6	73.1	71.46	72.1	73.11	56	59.5	60.01	61.15	59.50
7	72.7	70.86	68.7	72.99	57	59.5	60.17	62.15	59.50
8	72.7	70.92	76.2	72.98	58	61.1	59.95	63.748	61.10
9	72.7	70.91	75.8	72.69	59	61.1	60.07	64.75	61.10
10	69.4	69.75	72.3	69.38	60	61.1	60.25	65.42	61.10
11	69.4	69.95	75	69.38	61	60.8	60.03	66.45	60.80
12	69.4	70.09	70.8	69.40	62	60.8	60.20	62.165	60.80
13	70.5	69.07	66.2	70.57	63	60.8	60.45	65.125	60.80
14	70.5	69.29	65.2	70.57	64	60.5	59.91	65.15	61.34
15	70.5	69.42	74.8	70.50	65	60.5	59.88	68.15	61.29
16	68.1	68.38	75.4	68.10	66	60.5	59.95	54	60.50
17	68.1	68.60	69.55	68.10	67	59.9	59.92	61.226	58.64
18	68.1	68.75	74.6	68.10	68	59.9	59.91	60.4	58.71
19	67.8	67.98	63.3	67.80	69	59.9	59.99	60.8	59.90
20	67.8	68.13	70.3	67.80	70	57	59.67	56.1	57.42
21	67.8	68.19	63.5	67.80	71	57	59.74	58.65	57.40
22	67	66.78	61.7	66.99	72	57	59.95	59.65	57.00
23	67	66.82	71.3	66.99	73	59.7	60.13	62.348	60.75
24	67	66.78	74.3	67.00	74	59.7	60.00	63.35	60.69
25	64.1	65.15	65.55	64.10	75	59.7	59.92	61.1	59.70
26	64.1	65.16	63.1	64.10	76	60	59.78	66.5	58.41
27	64.1	65.14	60.1	64.10	77	60	59.70	55.5	58.50
28	64.6	65.09	68.1	64.57	78	60	59.68	62.5	59.99
29	64.6	64.63	67.7	64.57	79	59.6	58.72	57.95	60.14
30	64.6	64.26	67.5	64.60	80	59.6	58.72	55.1	60.11
31	64.4	64.83	70	64.46	81	59.6	58.83	57.95	59.60
32	64.4	64.51	65.8	64.46	82	62	62.71	63.32	62.36
33	64.4	64.31	70.9	64.40	83	62	63.11	64.56	61.68
34	64.7	64.32	60.2	64.70	84	62	63.36	66.36	62.01
35	64.7	64.23	69.2	64.70	85	62	61.70	66.32	61.31
36	64.7	64.16	61.2	64.70	86	62	61.75	61	60.70
37	63.9	64.34	68.4	63.90	87	62	61.67	58	61.95
38	63.9	63.96	61.9	63.90	88	60.6	60.12	64.1	60.99
39	63.9	63.57	67.4	63.90	89	60.6	59.90	63.7	61.05
40	63.4	63.31	67.6	63.39	90	60.6	59.56	63.5	60.62
41	63.4	63.20	65.05	63.39	91	62.1	61.54	67.7	61.53
42	63.4	63.07	58.1	63.40	92	62.1	61.98	66.6	61.34
43	62	62.46	66.6	62.00	93	62.1	62.25	58.6	62.10
44	62	62.61	64.5	62.00	94	61.5	60.58	66	62.30
45	62	62.59	60.35	62.00	95	61.5	60.90	59.5	61.99
46	62.4	62.92	57.9	62.16	96	61.5	61.06	65	61.49
47	62.4	62.88	60.75	62.17	97	57.8	59.01	62	57.57
48	62.4	62.81	63.72	62.40	98	57.8	59.27	59.45	57.82
49	61.7	61.38	64.26	62.09	99	57.8	59.38	52.5	57.81
50	61.7	61.74	66.06	62.09	100	61.5	61.79	66.1	62.12
101	61.5	61.93	65.82	61.96	151	47.3	47.75	48.7	47.30
102	61.5	61.92	67.15	61.51	152	47.3	47.39	43	47.30
103	60.8	60.13	62.165	59.97	153	47.3	47.31	42	47.30
104	60.8	60.23	65.125	59.81	154	52.1	51.48	56.4	52.06
105	60.8	60.22	65.45	60.78	155	52.1	50.55	59.4	51.62
106	57.6	57.83	65.25	57.47	156	52.1	49.77	53.55	51.44
107	57.6	57.95	51.1	57.55	157	45.5	47.91	52	45.78
108	57.6	57.99	59	57.60	158	45.5	47.32	41	46.21
109	58.8	59.18	54.5	58.80	159	45.5	46.90	48	45.69
110	58.8	58.83	53.5	58.80	160	45.7	45.70	41.4	45.70
111	58.8	58.72	63.1	58.86	161	45.7	45.40	40.4	45.81
112	56.8	57.14	64.1	56.09	162	45.7	45.15	50	45.59
113	56.8	57.15	58.25	56.66	163	49.6	49.51	45.6	49.39
114	56.8	57.29	63.3	56.70	164	49.6	49.60	51.25	50.05
115	55.3	55.73	50.8	55.14	165	49.6	49.64	52.25	50.12
116	55.3	55.73	57.8	55.34	166	48	49.09	50.648	48.45
117	55.3	55.67	51	55.32	167	48	48.93	51.65	48.19
118	57.8	56.73	52.5	57.80	168	48	48.74	49.4	48.29
119	57.8	56.76	62.1	57.81	169	47.7	48.56	54.2	47.47
120	57.8	56.82	65.1	57.79	170	47.7	48.24	43.2	47.31
121	56.6	56.38	58.05	56.71	171	47.7	47.91	50.2	47.42
122	56.6	56.59	55.6	56.61	172	49.1	49.29	47.45	49.01
123	56.6	56.62	52.6	56.62	173	49.1	48.99	44.6	48.91
124	56.9	55.43	58.55	56.79	174	49.1	48.61	47.45	48.83
125	56.9	55.36	59.55	56.88	175	48	47.99	49.32	47.90
126	56.9	54.98	59.548	56.89	176	48	48.09	50.56	47.89
127	56.1	55.66	59.75	56.10	177	48	48.10	50.9	47.97
128	56.1	56.38	57.5	56.32	178	48.5	47.45	54.1	48.46
129	56.1	56.85	62.6	56.33	179	48.5	47.49	53	48.24
130	55.9	56.10	51.4	55.90	180	48.5	47.42	46.5	48.31
131	55.9	56.72	58.4	55.55	181	49.4	49.37	48.4	49.77
132	55.9	56.92	54.25	55.54	182	49.4	49.34	45.4	50.25
133	54.3	54.90	49.8	54.30	183	49.4	49.21	52.9	50.28
134	54.3	55.07	52.65	54.43	184	48.7	47.73	51.8	48.31
135	54.3	54.79	55.62	54.40	185	48.7	47.74	51.6	48.30
136	54.2	54.71	56.76	54.20	186	48.7	47.65	54.3	48.36
137	54.2	54.72	57.1	54.20	187	46.1	46.38	47.5	46.47
138	54.2	54.98	59.8	54.20	188	46.1	46.41	41.8	46.45
139	52.7	52.73	57.2	52.77	189	46.1	46.36	40.8	46.53
140	52.7	52.59	49.2	52.73	190	47.7	46.08	52	46.63
141	52.7	52.75	57.2	52.72	191	47.7	46.49	55	46.60
142	51	49.93	49	50.94	192	47.7	46.99	49.15	46.57
143	51	49.83	54.5	50.97	193	47.1	47.69	53.6	48.46
144	51	50.09	55.2	50.98	194	47.1	47.99	42.6	48.54
145	54.6	53.14	56.25	54.57	195	47.1	48.39	49.6	48.62
146	54.6	52.50	49.3	54.22	196	45	45.16	40.7	44.99
147	54.6	52.13	59.2	54.07	197	45	45.46	47.648	44.98
148	50.3	50.46	54.62	50.33	198	45	45.69	48.65	44.97
149	50.3	49.83	53.2	50.68	199	46	45.99	47.4	46.04
150	50.3	49.49	55.9	50.83	200	46	46.37	52.5	46.06
201	46	46.66	41.5	46.08	251	43.7	44.64	45.02	40.90
202	45.7	45.41	48.2	45.57	252	43.7	44.44	46.26	41.08
203	45.7	45.67	44.05	45.53	253	44.5	43.58	48.86	42.61
204	45.7	45.77	41.2	45.49	254	44.5	43.64	48.82	41.67
205	45.1	44.54	43.45	45.18	255	44.5	43.70	43.5	41.81
206	45.1	44.59	47.748	45.21	256	42.6	43.74	38.6	50.61
207	45.1	44.47	48.75	45.24	257	42.6	43.72	46.1	47.70
208	46	45.83	50.32	46.00	258	42.6	43.70	45.7	47.80
209	46	46.23	51.65	46.00	259	43.8	43.80	46.7	50.12
210	46	46.42	47.365	46.00	260	43.8	43.71	49.4	47.82
211	45	44.59	49.325	45.00	261	43.8	43.58	39.8	47.91
212	45	44.74	49.65	45.00	262	43.6	43.39	47.1	41.74
213	45	44.69	52.65	45.00	263	43.6	43.33	46.7	41.33
214	43.3	42.93	36.8	43.30	264	43.6	43.24	46.5	41.45
215	43.3	42.86	44.626	43.31	265	42.6	43.00	48.2	40.71
216	43.3	42.65	43.8	43.32	266	42.6	42.95	44	39.51
217	44.5	45.50	45.4	44.55	267	42.6	42.86	38.3	39.68
218	44.5	45.37	43.6	44.55	268	42.9	42.61	37.6	42.12
219	44.5	45.45	46.15	44.55	269	42.9	42.55	47.2	41.22
220	43.6	43.97	46.25	43.47	270	42.9	42.43	50.2	41.36
221	43.6	44.02	38.3	43.46	271	44.9	42.59	46.35	38.73
222	43.6	44.29	47.9	43.45	272	44.9	43.09	51.4	38.82
223	42	43.28	49.3	42.08	273	44.9	43.56	40.4	38.85
224	42	43.50	43.45	42.09	274	41.1	41.81	43.6	37.85
225	42	43.92	41	42.10	275	41.1	42.18	36.8	38.19
226	43.8	43.83	39.8	43.80	276	41.1	42.52	35.8	38.53
227	43.8	43.59	45.45	41.45	277	41.5	41.30	44.148	38.22
228	43.8	43.55	46.45	39.12	278	41.5	41.52	45.15	38.56
229	43	43.11	45.648	39.02	279	41.5	41.72	42.9	38.90
230	43	43.04	46.65	37.72	280	42.5	42.57	49	36.86
231	43	43.14	44.4	36.43	281	42.5	42.93	38	36.61
232	43.2	42.79	49.7	34.85	282	42.5	43.21	45	36.35
233	43.2	42.77	38.7	33.75	283	40.8	41.94	39.15	36.25
234	43.2	42.85	45.7	32.65	284	40.8	42.09	36.3	35.84
235	43.5	43.17	41.85	32.82	285	40.8	42.17	39.15	35.44
236	43.5	42.85	39	33.69	286	40.8	41.04	43.448	37.12
237	43.5	42.67	41.85	34.56	287	40.8	41.03	44.45	36.75
238	41.5	42.54	42.82	34.82	288	40.8	40.98	45.12	36.38
239	41.5	42.32	44.06	34.96	289	41.8	42.54	47.45	33.92
240	41.5	42.17	44.4	35.10	290	41.8	42.63	43.165	34.80
241	42.4	41.81	48	36.10	291	41.8	42.66	46.125	35.67
242	42.4	41.57	46.9	35.69	292	41.3	41.52	45.95	33.34
243	42.4	41.32	46.05	35.29	293	41.3	41.42	48.95	34.22
244	46	44.86	47.4	45.53	294	41.3	41.28	34.8	35.09
245	46	44.84	52.5	45.02	295	41	40.25	42.326	34.05
246	46	44.83	41.5	45.15	296	41	40.06	41.5	34.82
247	45	44.92	47.5	42.38	297	41	39.87	41.9	35.59
248	45	44.78	43.35	40.90	298	41.3	41.50	40.4	37.73
249	45	44.66	40.5	41.08	299	41.3	41.58	42.95	37.69
250	43.7	44.88	42.05	42.38	300	41.3	41.78	43.95	37.64
301	41.5	40.78	36.2	37.07					
302	41.5	40.90	45.8	37.00					
303	41.5	41.11	44	36.92					
304	40.3	40.02	38.65	36.07					
305	40.3	40.10	35.8	35.99					
306	40.3	40.21	38.65	35.91					
307	41.5	41.23	42.82	33.84					
308	41.5	41.25	44.06	29.97					
309	41.5	41.35	44.4	26.11					
310	40	40.59	45.6	29.15					
311	40	40.59	44.5	26.80					
312	40	40.62	43.65	24.46					
313	40	39.77	41.4	25.04					
314	40	39.68	46.5	23.55					
315	40	39.58	35.5	22.06					
316	42.1	40.82	44.6	27.86					
317	42.1	40.69	40.45	27.03					
318	42.1	40.60	37.6	26.20					
319	39.5	40.02	37.85	28.95					
320	39.5	39.85	47.15	28.12					
321	39.5	39.70	33	27.30					
322	37.5	39.13	38.826	30.77					
323	37.5	38.95	38	29.45					
324	37.5	38.79	38.4	28.12					
