# Supplementary material for: Predicting High-Strength Concrete’s Compressive Strength: A Comparative Study of Artificial Neural Networks, Adaptive Neuro-Fuzzy Inference System, and Response Surface Methodology
Source: Materials (Basel). 2024 Sep 15;17(18):4533. doi: 10.3390/ma17184533 (PMC11432809; doi:10.3390/ma17184533)
Supplement: Supplementary file 1 [file materials-17-04533-s001.zip › materials-3146923-supplementary.pdf]

Predicting High Strength Concrete Compressive Strength: A Comparative Study of Artificial Neural Networks, Adaptive Neuro-Fuzzy Inference System, and Response Surface Methodology

Supplementary Materials:

Table S1. The experimental dataset [40], [41].

ID	Water	OPC	FA	CA	SP	Actual compressive strength
	(kg/m3)	(kg/m3)	(kg/m3)	(kg/m3)	(kg/m3)	Mpa
1	160	533	805	845	1	73.6
2	160	533	805	845	1.5	73.6
3	160	533	805	845	2	73.6
4	160	480	786	845	1	73.1
5	160	480	786	845	1.5	73.1
6	160	480	786	845	2	73.1
7	160	427	767	845	1	72.7
8	160	427	767	845	1.5	72.7
9	160	427	767	845	2	72.7
10	160	533	753	898	1	69.4
11	160	533	753	898	1.5	69.4
12	160	533	753	898	2	69.4
13	160	480	734	898	1	70.5
14	160	480	734	898	1.5	70.5
15	160	480	734	898	2	70.5
16	160	427	715	898	1	68.1
17	160	427	715	898	1.5	68.1
18	160	427	715	898	2	68.1
19	160	533	701	950	1	67.8
20	160	533	701	950	1.5	67.8
21	160	533	701	950	2	67.8
22	160	480	682	950	1	67
23	160	480	682	950	1.5	67
24	160	480	682	950	2	67
25	160	427	663	950	1	64.1
26	160	427	663	950	1.5	64.1
27	160	427	663	950	2	64.1
28	170	567	751	845	1	64.6
29	170	567	751	845	1.5	64.6
30	170	567	751	845	2	64.6
31	170	510	731	845	1	64.4

32	170	510	731	845	1.5	64.4
33	170	510	731	845	2	64.4
34	170	453	711	845	1	64.7
35	170	453	711	845	1.5	64.7
36	170	453	711	845	2	64.7
37	170	567	700	898	1	63.9
38	170	567	700	898	1.5	63.9
39	170	567	700	898	2	63.9
40	170	510	679	898	1	63.4
41	170	510	679	898	1.5	63.4
42	170	510	679	898	2	63.4
43	170	453	659	898	1	62
44	170	453	659	898	1.5	62
45	170	453	659	898	2	62
46	170	567	648	950	1	62.4
47	170	567	648	950	1.5	62.4
48	170	567	648	950	2	62.4
49	170	510	628	950	1	61.7
50	170	510	628	950	1.5	61.7
51	170	510	628	950	2	61.7
52	170	453	608	950	1	61.9
53	170	453	608	950	1.5	61.9
54	170	453	608	950	2	61.9
55	180	600	698	845	0.75	59.5
56	180	600	698	845	1.25	59.5
57	180	600	698	845	1.75	59.5
58	180	540	677	845	0.75	61.1
59	180	540	677	845	1.25	61.1
60	180	540	677	845	1.75	61.1
61	180	480	655	845	0.75	60.8
62	180	480	655	845	1.25	60.8
63	180	480	655	845	1.75	60.8
64	180	600	646	898	0.75	60.5
65	180	600	646	898	1.25	60.5
66	180	600	646	898	1.75	60.5
67	180	540	625	898	0.75	59.9
68	180	540	625	898	1.25	59.9
69	180	540	625	898	1.75	59.9
70	180	480	604	898	0.75	57
71	180	480	604	898	1.25	57
72	180	480	604	898	1.75	57
73	180	600	594	950	0.75	59.7
74	180	600	594	950	1.25	59.7
75	180	600	594	950	1.75	59.7
76	180	540	573	950	0.75	60

77	180	540	573	950	1.25	60
78	180	540	573	950	1.75	60
79	180	480	552	950	0.75	59.6
80	180	480	552	950	1.25	59.6
81	180	480	552	950	1.75	59.6
82	160	457	867	845	0.75	62
83	160	457	867	845	1.25	62
84	160	457	867	845	1.75	62
85	160	411	851	845	0.75	62
86	160	411	851	845	1.25	62
87	160	411	851	845	1.75	62
88	160	366	835	845	0.75	60.6
89	160	366	835	845	1.25	60.6
90	160	366	835	845	1.75	60.6
91	160	457	816	898	0.75	62.1
92	160	457	816	898	1.25	62.1
93	160	457	816	898	1.75	62.1
94	160	411	799	898	0.75	61.5
95	160	411	799	898	1.25	61.5
96	160	411	799	898	1.75	61.5
97	160	366	783	898	0.75	57.8
98	160	366	783	898	1.25	57.8
99	160	366	783	898	1.75	57.8
100	160	457	764	950	0.75	61.5
101	160	457	764	950	1.25	61.5
102	160	457	764	950	1.75	61.5
103	160	411	747	950	0.75	60.8
104	160	411	747	950	1.25	60.8
105	160	411	747	950	1.75	60.8
106	160	366	731	950	0.75	57.6
107	160	366	731	950	1.25	57.6
108	160	366	731	950	1.75	57.6
109	170	486	818	845	0.5	58.8
110	170	486	818	845	1	58.8
111	170	486	818	845	1.5	58.8
112	170	437	801	845	0.5	56.8
113	170	437	801	845	1	56.8
114	170	437	801	845	1.5	56.8
115	170	389	783	845	0.5	55.3
116	170	389	783	845	1	55.3
117	170	389	783	845	1.5	55.3
118	170	486	766	898	0.5	57.8
119	170	486	766	898	1	57.8
120	170	486	766	898	1.5	57.8
121	170	437	749	898	0.5	56.6

122	170	437	749	898	1	56.6
123	170	437	749	898	1.5	56.6
124	170	389	732	898	0.5	56.9
125	170	389	732	898	1	56.9
126	170	389	732	898	1.5	56.9
127	170	486	714	950	0.5	56.1
128	170	486	714	950	1	56.1
129	170	486	714	950	1.5	56.1
130	170	437	697	950	0.5	55.9
131	170	437	697	950	1	55.9
132	170	437	697	950	1.5	55.9
133	170	389	680	950	0.5	54.3
134	170	389	680	950	1	54.3
135	170	389	680	950	1.5	54.3
136	180	514	769	845	0.25	54.2
137	180	514	769	845	0.75	54.2
138	180	514	769	845	1.25	54.2
139	180	463	750	845	0.25	52.7
140	180	463	750	845	0.75	52.7
141	180	463	750	845	1.25	52.7
142	180	411	732	845	0.25	51
143	180	411	732	845	0.75	51
144	180	411	732	845	1.25	51
145	180	514	717	898	0.25	54.6
146	180	514	717	898	0.75	54.6
147	180	514	717	898	1.25	54.6
148	180	463	698	898	0.25	50.3
149	180	463	698	898	0.75	50.3
150	180	463	698	898	1.25	50.3
151	180	411	680	898	0.25	47.3
152	180	411	680	898	0.75	47.3
153	180	411	680	898	1.25	47.3
154	180	514	665	950	0.25	52.1
155	180	514	665	950	0.75	52.1
156	180	514	665	950	1.25	52.1
157	180	463	647	950	0.5	45.5
158	180	463	647	950	1	45.5
159	180	463	647	950	1.5	45.5
160	180	411	628	950	0.5	45.7
161	180	411	628	950	1	45.7
162	180	411	628	950	1.5	45.7
163	160	400	914	845	0.5	49.6
164	160	400	914	845	1	49.6
165	160	400	914	845	1.5	49.6
166	160	360	900	845	0.5	48

167	160	360	900	845	1	48
168	160	360	900	845	1.5	48
169	160	320	886	845	0.5	47.7
170	160	320	886	845	1	47.7
171	160	320	886	845	1.5	47.7
172	160	400	863	989	0.5	49.1
173	160	400	863	989	1	49.1
174	160	400	863	989	1.5	49.1
175	160	360	848	898	0.5	48
176	160	360	848	898	1	48
177	160	360	848	898	1.5	48
178	160	320	834	898	0.5	48.5
179	160	320	834	898	1	48.5
180	160	320	834	898	1.5	48.5
181	160	400	811	950	0.5	49.4
182	160	400	811	950	1	49.4
183	160	400	811	950	1.5	49.4
184	160	360	797	950	0.5	48.7
185	160	360	797	950	1	48.7
186	160	360	797	950	1.5	48.7
187	160	320	782	950	0.5	46.1
188	160	320	782	950	1	46.1
189	160	320	782	950	1.5	46.1
190	170	425	868	845	0	47.7
191	170	425	868	845	0.5	47.7
192	170	425	868	845	1	47.7
193	170	425	853	845	0	47.1
194	170	425	853	845	0.5	47.1
195	170	425	853	845	1	47.1
196	170	340	838	845	0	45
197	170	340	838	845	0.5	45
198	170	340	838	845	1	45
199	170	425	816	898	0	46
200	170	425	816	898	0.5	46
201	170	425	816	898	1	46
202	170	383	801	898	0	45.7
203	170	383	801	898	0.5	45.7
204	170	383	801	898	1	45.7
205	170	340	786	898	0	45.1
206	170	340	786	898	0.5	45.1
207	170	340	786	898	1	45.1
208	170	425	764	950	0	46
209	170	425	764	950	0.5	46
210	170	425	764	950	1	46
211	170	383	749	950	0	45

212	170	383	749	950	0.5	45
213	170	383	749	950	1	45
214	170	340	734	950	0	43.3
215	170	340	734	950	0.5	43.3
216	170	340	734	950	1	43.3
217	180	450	821	845	0	44.5
218	180	450	821	845	0.5	44.5
219	180	450	821	845	1	44.5
220	180	405	805	845	0	43.6
221	180	405	805	845	0.5	43.6
222	180	405	805	845	1	43.6
223	180	360	789	845	0	42
224	180	360	789	845	0.5	42
225	180	360	789	845	1	42
226	180	450	770	898	0	43.8
227	180	450	770	898	0.5	43.8
228	180	450	770	898	1	43.8
229	180	405	754	898	0	43
230	180	405	754	898	0.5	43
231	180	405	754	898	1	43
232	180	360	738	898	0	43.2
233	180	360	738	898	0.5	43.2
234	180	360	738	898	1	43.2
235	180	450	718	950	0	43.5
236	180	450	718	950	0.5	43.5
237	180	450	718	950	1	43.5
238	180	405	702	950	0	41.5
239	180	405	702	950	0.5	41.5
240	180	405	702	950	1	41.5
241	180	360	686	950	0	42.4
242	180	360	686	950	0.5	42.4
243	180	360	686	950	1	42.4
244	160	356	951	845	0.5	46
245	160	356	951	845	1	46
246	160	356	951	845	1.5	46
247	160	320	938	845	0.5	45
248	160	320	938	845	1	45
249	160	320	938	845	1.5	45
250	160	284	926	845	0.5	43.7
251	160	284	926	845	1	43.7
252	160	284	926	845	1.5	43.7
253	160	356	899	898	0.5	44.5
254	160	356	899	898	1	44.5
255	160	356	899	898	1.5	44.5
256	160	320	886	898	0.5	42.6

257	160	320	886	898	1	42.6
258	160	320	886	898	1.5	42.6
259	160	284	874	898	0.5	43.8
260	160	284	874	898	1	43.8
261	160	284	874	898	1.5	43.8
262	160	356	847	950	0.5	43.6
263	160	356	847	950	1	43.6
264	160	356	847	950	1.5	43.6
265	160	320	835	950	0.5	42.6
266	160	320	835	950	1	42.6
267	160	320	835	950	1.5	42.6
268	160	284	822	950	0.5	42.9
269	160	284	822	950	1	42.9
270	160	284	822	950	1.5	42.9
271	170	378	907	845	0.5	44.9
272	170	378	907	845	1	44.9
273	170	378	907	845	1.5	44.9
274	170	340	893	845	0	41.1
275	170	340	893	845	0.5	41.1
276	170	340	893	845	1	41.1
277	170	302	880	845	0	41.5
278	170	302	880	845	0.5	41.5
279	170	302	880	845	1	41.5
280	170	378	855	898	0	42.5
281	170	378	855	898	0.5	42.5
282	170	378	855	898	1	42.5
283	170	340	842	898	0	40.8
284	170	340	842	898	0.5	40.8
285	170	340	842	898	1	40.8
286	170	302	828	898	0	40.8
287	170	302	828	898	0.5	40.8
288	170	302	828	898	1	40.8
289	170	378	803	950	0	41.8
290	170	378	803	950	0.5	41.8
291	170	378	803	950	1	41.8
292	170	340	790	950	0	41.3
293	170	340	790	950	0.5	41.3
294	170	340	790	950	1	41.3
295	170	302	776	950	0	41
296	170	302	776	950	0.5	41
297	170	302	776	950	1	41
298	180	400	863	845	0	41.3
299	180	400	863	845	0.5	41.3
300	180	400	863	845	1	41.3
301	180	360	848	845	0	41.5

302	180	360	848	845	0.5	41.5
303	180	360	848	845	1	41.5
304	180	320	834	845	0	40.3
305	180	320	834	845	0.5	40.3
306	180	320	834	845	1	40.3
307	180	400	811	898	0	41.5
308	180	400	811	898	0.5	41.5
309	180	400	811	898	1	41.5
310	180	360	797	898	0	40
311	180	360	797	898	0.5	40
312	180	360	797	898	1	40
313	180	320	782	898	0	40
314	180	320	782	898	0.5	40
315	180	320	782	898	1	40
316	180	400	759	950	0	42.1
317	180	400	759	950	0.5	42.1
318	180	400	759	950	1	42.1
319	180	360	745	950	0	39.5
320	180	360	745	950	0.5	39.5
321	180	360	745	950	1	39.5
322	180	320	731	950	0	37.5
323	180	320	731	950	0.5	37.5
324	180	320	731	950	1	37.5
